# Supplementary material for: Direct Base-Assisted C‒H Cyclonickelation of 6-Phenyl-2,2′-bipyridine
Source: Molecules. 2020 Feb 24;25(4):997. doi: 10.3390/molecules25040997 (PMC7070369; doi:10.3390/molecules25040997)
Supplement: Supplementary file 1 [file molecules-25-00997-s001.zip › molecules-724802-supplementary.pdf]

Supplementary Materials

Direct C–H Cyclonickelation of 6-Phenyl-2,2′-bipyridine

Nicolas Vogt, Vasily Sivchik, Aaron Sandleben, Gerald Hörner, Axel Klein*

Contents

1. EXPERIMENTAL SECTION

2. SUPPORTING FIGURES AND TABLES
Figure S1. 600 MHz ^{1}H NMR spectrum of [Ni(Phbpy)Br] in CD_2Cl_2.
Figure S2. 400 MHz ^{1}H NMR spectrum of [Ni(Phbpy)Br] in CD_2Cl_2.
Figure S3. 400 MHz ^{1}H NMR spectrum of [Ni(Phbpy)Br] in acetone-d^6.
Figure S4. 400 MHz ^{1}H-^{1}H COSY spectrum of [Ni(Phbpy)Br] in acetone-d^6.
Figure S5. UV-vis absorption spectra recorded during the reaction of $[Ni(HPhbpy)Br_2]_2$ in *p*-xylene in the presence of KOAc/K_2CO_3.
Figure S6. Molecular structure of [Ni(Phbpy)(CN)] (left) and crystal structure viewed along the *c* axis (right). Note that the structure could not be solved satisfactorily. These plots show only preliminary results.
Table S1. Parameters for the optimisation of the cyclonickelation of HPhbpy.
Table S2. Crystal data, refinement parameters, and important bond parameters of the structure of the binuclear complex $[Ni(HPhbpy)Br_2]_2$.
Table S3. Crystal data, structure refinement, and important bond parameters for [Ni(Phbpy)(OAc)].
Table S4. Crystal data for [Ni(Phbpy)(CN)].

3. REFERENCES

1. EXPERIMENTAL SECTION

Preparation of *N*-(1-(2-pyridyl)-1-oxo-2-ethyl)pyridinium-iodide (*Kroehnke* salt): 12.1 g 2-acetylpyridine (11.2 mL, 100 mmol, 1 eq.) and 25.4 g iodine (100 mmol, 1 eq.) were refluxed in 120 mL of pyridine for 1 h. After cooling to room temperature the reaction mixture was filtered and the resulting dark-grey precipitate was washed with a small amount of cold pyridine and then acetone and dried in vacuum. Yield: 21.98 g, 71.0 mmol, 71%. ^{1}H NMR (300 MHz, DMSO-d_6) δ = 9.04-8.96 (d, 2H, J = 5.2 Hz), 8.91-8.83 (d, 1H, J = 4.5 Hz), 8.78-8.66 (t, 1H, J = 7.8 Hz), 8.32-8.21 (dd, 2H, J = 7.9, 6.6 Hz), 8.17-8.02 (m, 2H), 7.89-7.77 (ddd, 1H, J = 7.4, 4.8, 1.6 Hz), 6.51 (s, 2H) ppm.

Preparation of 6-Phenyl-2,2'-bipyridine (HPhbpy): The *Kroehnke* salt (6.9 g, 21 mmol, 1 eq.) and ammonium acetate (16.3 g, 210 mmol, 10 eq.) were suspended in glacial acetic acid (58 mL) and heated under reflux for 30 min. After the addition of the *Mannich* adduct the mixture was further heated under refluxed for 22 h. After cooling to room temperature, the solvent was reduced as far as possible. The oily residue was diluted with 100 mL of chloroform and stirred with 100 mL of water. The precipitated black solid was filtered off and the organic layer was washed with 100 mL of a saturated sodium carbonate solution and afterwards two times with 100 mL of water. After drying over magnesium sulphate the solvent was evaporated and the oily raw product was purified by filtration over silica (cyclohexane/ethyl acetate 9/1). The product was dried in vacuum and was received as a bright brown powder. Yield: 54% (3.22 g, 13.86 mmol). ^{1}H NMR (300 MHz, $CDCl_3$) δ = 8.69 (d, 1H, J_{HH} = 4.6 Hz), 8.64 (d, 1H, J_{HH} = 8.0 Hz), 8.38 (d, 1H, J_{HH} = 7.8 Hz), 8.15 (d, 1H, J_{HH} = 7.8 Hz), 7.97-7.75 (m, 3H), 7.53-7.41 (m, 3H), 7.31 (t, 1H, J_{HH} = 6.1 Hz) ppm. ^{13}C NMR: (75 MHz, $CDCl_3$) δ = 156.6, 156.5, 155.9, 149.2, 139.5, 137.8, 137.0, 129.1, 128.9, 127.1, 123.9, 121.4, 120.4, 119.4 ppm. Elemental analysis found (calc. for $C_{16}H_{12}N_2$): C 82.71 (82.73); H 5.17 (5.16); N 11.92 (11.90)%.

General Information on the C–H activation reactions (direct nickelation)

Diethyl ether, toluene, and tetrahydrofuran (THF) were distilled over sodium benzophenone ketyl under a nitrogen atmosphere prior to use. $NiBr_2$ was a reagent grade and used as received. To obtain anhydrous nickel(II) acetate, the tetrahydrate $Ni(OAc)_2{\cdot}4H_2O$ was dried at 150°C under vacuum for 20 h. The obtained materials were stored in a glovebox. Unless otherwise stated other chemicals and solvents were received from commercial suppliers and used without the purification. ^1H and ^1H-^1H COSY NMR spectra were recorded on a Bruker Avance 400 spectrometer. Microanalysis was carried out at the analytical laboratory of the University of Eastern Finland.

Details on the cyclonickelation using $NiBr_2$

Table S1. Parameters for the optimisation of the cyclonickelation of HPhbpy.

entry	method [a]	NiX_2	base (eq.)	solvent (conc.)	time (h)	*T* (°C)	mode	yield (%)	analysis
**1**	A	$NiBr_2$	no base	toluene (20 mM→solid)	60	111	reflux [c]	0	visual [b]
**2**	A	$NiBr_2$	NEt_3 (11.5)	toluene (20 mM)	48	111	reflux [d]	1.5	^1H NMR
**3**	A	$NiBr_2$	$NaHCO_3$ (2)	toluene/MeOH (5/2 v/v; 3.6→5.6 mM)	3	111	Dean-Stark	2.5	^1H NMR
**4**	A	$NiBr_2$	Na_2CO_3 (4)	-	1	170	solid	0	visual [b]
**5**	B	$NiBr_2$	Cs_2CO_3 (2)	toluene (11.4 mM)	66	111	reflux [e]	trace (recryst.)	^1H NMR
**6**	B	$NiBr_2$	Na_2CO_3 (3)	toluene (10mM→100mM)	90	111	reflux [g]	**19 (crude)** 2 (recryst.)	^1H NMR
**7**	C	$[Ni(HPhbpy)Br_2]_2$	NaO*t*Bu (1)	diethyl ether	16	23	stirring	0	^1H NMR
**8**	C	$[Ni(HPhbpy)Br_2]_2$	KOAc (2) K_2CO_3 (4)	toluene (4 mM)	16	111	Dean-Stark	6 (recryst.)	^1H NMR
**9**	C	$[Ni(HPhbpy)Br_2]_2$	KOAc (2) K_2CO_3 (4)	toluene (3.5 mM)	62	111	Dean-Stark	**28 (recryst.)**	^1H NMR
**10**	A	$Ni(OAc)_2{\cdot}4H_2O$	-	toluene/EtOH/DME (1:1.3:8 v/v/v, 3.2 mM)	15	111	Dean-Stark	5 (recryst.) [g]	XRD
**11**	A	$Ni(OAc)_2$ dried	-	-	1	250	solid	0 [h]	visual [b]
**12**	A	$Ni(OAc)_2$ dried	-	-	1	180	solid	4 [i]	XRD
**13**	C	$[Ni(HPhbpy)Br_2]_2$	KOAc (2) K_2CO_3 (4)	toluene	72	120	Dean-Stark	43	UV-vis [k]
**14**	C	$[Ni(HPhbpy)Br_2]_2$	KOAc (2) K_2CO_3 (4)	chlorobenzene	64	160	Dean-Stark	91	UV-vis [k]
**15**	C	$[Ni(HPhbpy)Br_2]_2$	KOAc (2) K_2CO_3 (4)	1,2-dichlorobenzene	25	190	Dean-Stark	98	UV-vis [k]
**16**	C	$[Ni(HPhbpy)Br_2]_2$	KOAc (2) K_2CO_3 (4)	*p*-xylene	25	180	Dean-Stark	100	UV-vis [k]
**17**	C	$[Ni(HPhbpy)Br_2]_2$	KOAc (2) K_2CO_3 (4)	benzonitrile	20 / 60	200	Dean-Stark	0	UV-vis [k]
**18**	B	$NiBr_2$	KOAc (1) K_2CO_3 (2)	*p*-xylene	25	180	Dean-Stark	100	UV-vis [k]
**19**	C	$[Ni(HPhbpy)Br_2]_2$	no base	1,2-dichlorobenzene	64	190	Dean-Stark	15	UV-vis [k]

[a] **Method A:** strictly direct cyclonickelation using $NiBr_2$ or $Ni(OAc)_2$. **Method B:** cyclonickelation via *in-situ* formation of $[Ni(HPhbpy)Br_2]_2$. **Method C:** starting from isolated $[Ni(HPhbpy)Br_2]_2$. [b] The target complex has a characteristic red colour and can be easily traced visually in the reaction solution by the naked eye. [c] Solvent evaporated in reflux. [d] Reagents were homogenised in CH_2Cl_2/MeOH. [e] Recrystallised from CH_2Cl_2/Et_2O. [f] Recrystallised from CH_2Cl_2/Et_2O and acetone/Et_2O. [g] [Ni(Phbpy)(OAc)] was obtained. [h] Red material recrystallised from HOAc gave green-grey decomposed material. [i] Solid mixture heated while evaporated, trapped by NaCN in THF and recrystallised from CH_2Cl_2, yielding [Ni(Phbpy)(CN)]. [k] Yields as observed from UV-vis absorption measurements of the reaction solutions, based on the reported molar

extinction coefficients of the long-wavelength maximum of the target complex [Ni(Phbpy)Br] at around 510 nm.

Entry 1. $NiBr_2$ (110 mg, 0.50 mmol) and HPhbpy (123 mg, 0.53 mmol) were put into a Schlenk flask and the mixture was evacuated for 30 min at ca. 50°C and filled with nitrogen. Then, distilled toluene (ca. 25 mL) was added and the mixture was heated under reflux for 60 h. The solvent completely evaporated during this time giving a green-yellow product mixture and no indication for the red cyclometalated product.

Entry 2. The procedure is performed as a modified version of ref.[8] $NiBr_2$ (219 mg, 1 mmol) in CH_3OH (16 mL) was added to the solution of HPhbpy (223 mg, 0.96 mmol) in CH_2Cl_2/CH_3OH mixture (20 mL, 1:1 v/v). Distilled toluene (ca. 50 mL) was added and the mixture was stirred for 16 h at ambient temperature. The solvents were evaporated; the grey-green solid was suspended in nitrogen-purged toluene (50 mL) and triethylamine (1.6 mL, 1161 mg, 11.5 mmol). The mixture was heated under reflux for 48 h. A red solution is formed. Filtration and evaporation of the solution gave a red solid which was washed using pentane (2 x 4 mL). Yield: 5.3 mg (1.5%). ^{1}H NMR (Figure S1) corresponds to the targeted complex [Ni(Phbpy)Br].[6] In CD_3OD solution the compound slowly hydrolysed with a colour change from red to green and the ^{1}H NMR spectrum shows markedly broadened signals.

Entry 3. $NiBr_2$ (110 mg, 0.50 mmol) and HPhbpy (123 mg, 0.53 mmol) in CH_3OH (25 mL) were heated under reflux for 16 h resulting in a green solution. Freshly milled $NaHCO_3$ (84 mg, 1 mmol) and CH_3OH (15 mL) were added, the former is not completely dissolved. The mixture was transferred by cannula into distilled toluene (ca. 100 mL). Freshly milled $NaHCO_3$ (42 mg, 0.5 mmol) was added and the mixture was heated to 160°C. Solvents (90 mL) were collected into Dean-Stark trap in 30 min. Then the mixture was refluxed for 3 h. The colour change of the suspension from yellow to red was observed. The solvents were completely evaporated in vacuo. Distilled THF (50 mL) was added. The mixture was stirred for 10 min, filtered over Celite, evaporated in the Schlenk flask. The solid was washed at air by pentane (3×4 mL), hexane (1×4 mL), *n*-pentane (1×4 mL). **Yield: 5 mg (2,5%)** of a red solid. The washings were evaporated yielding a white solid (120 mg). ^{1}H NMR ($CDCl_3$) corresponds to unreacted HPhbpy.

Entry 4. (adopted from ref. [9] $NiBr_2$ (109 mg, 0.50 mmol), HPhbpy (116 mg, 0.50 mmol) anhydrous Na_2CO_3 (245 mg, 2.31 mmol) were milled in the mortar over 1 min. The pale-yellow powder was transferred (via funnel) to the 25-mL Schlenk flask. Under nitrogen the flask with the powder was immersed into oil bath at 130°C. No colour change was noticed, and the oil bath was heated to 170 °C during 40 min. (ca. 10°C increment every 10 min). The colour changed to some green. The vacuum was applied, and the powder was kept at this temperature for 1.5 h. The final solid colour is beige (indicating the absence of cyclometalation). No red solution was observed upon the addition of CH_2Cl_2.

Entry 5. Into a flame-dried 100 mL Schlenk flask $NiBr_2$, (55 mg, 0.25 mmol) was charged and dissolved in THF (30 mL) under heating. The residual yellow solid was eliminated by filtration. To this violet/pink solution HPhbpy (58 mg, 0.25 mmol) in THF (20 mL) was added under stirring. Within one minute the yellow solid started to precipitate. The suspension was stirred for 30 min. The yellow solid was collected by cannula filtration and dried using a flow of nitrogen for 10 min and in vacuo for 30 min. A small amount (ca. 3 mg) was taken for microanalysis. The intermediate yellow solid precipitated in THF and was shown to be [Ni(HPhbpy)Br_2] by elemental analysis. Anal. calc. for $C_{16}H_{12}Br_2N_2Ni$: C, 42.63; H, 2.68; N, 6.21%. Found: C, 42.67; H, 2.66; N, 6.24%.

To the *in situ* generated [Ni(HPhbpy)Br_2]$_2$ freshly milled Cs_2CO_3 (123 mg, 0.5 mmol) and toluene (22 mL) were added. The mixture was refluxed for 66 h. The suspension turned to red. Upon cooling some yellow and white solids become visible. The solvent was evaporated *in vacuo*. The beige solid was suspended in THF (80 mL). THF red solution was obtained by filtration on Celite. After the evaporation in vacuo the reddish paste-like substance with some white inclusions was obtained. It was dissolved in CH_2Cl_2 (10 mL) and crystallised by vapour diffusion of Et_2O at r.t. After 4 days red crystals were collected and washed by

Et_2O (3 × 1 mL), quickly by CH_3OH (2 × 1 mL; dissolves yellow powder), Et_2O (2 × 1 mL). **Yield: 2% (2.8 mg).** ^1H NMR in CD_2Cl_2 shows the expected compound, but broad signals. Red blocks were crystallised over 2 days by vapour diffusion of Et_2O to this NMR sample at r.t.

Entry 6. $NiBr_2$ (109 mg, 0.50 mmol) and HPhbpy (116 mg, 0.50 mmol) in THF (60 mL) were heated under reflux for 15 h. The yellow solid paste solid was filtered via cannula and dried in vacuo for 1 h. Milled anhydrous Na_2CO_3 (159 mg, 1.5 mmol) and toluene (50 mL) were added. The reaction was refluxed for 2 h. Further, it was refluxed using Dean-Stark head for 88 h. The first 3 mL of toluene was immediately collected. After 16 h and 40 h, 5 mL of toluene were collected each time. At the end of the reflux the mixture was concentrated to ca. 5 mL of the residual volume by distilling the solvent to Dean-Stark head. THF (50 mL) was added, stirred for 2 h. The yellow solid was eliminated by filtration over Celite 545 pad. The solution was evaporated yielding the crude red material. **Yield: 70 mg (19%)**.
The red solid (ca. 60 % of all material) was dissolved in CH_2Cl_2 (15 mL) and crystallised by vapour diffusion of Et_2O at r.t. After 4 days, the red crystals were collected washed by Et_2O (3×2 mL), quickly by CH_3OH (2×2 mL), Et_2O (2×2 mL). **Yield: 2.8 mg (1.5%)**. ^1H NMR in CD_2Cl_2 shows the target compound. Anal. calc. for $C_{16}H_{11}BrN_2Ni$: C, 51.96; H, 3.00; N, 7.57. Found: C, 51.69; H, 3.15; N, 7.64%.
Another recrystallisation (acetone/ether) gave red crystals, which were washed quickly using CH_3OH (2 × 0.5 mL), Et_2O (2 × 1 mL). **Yield: 1 mg (1%)**. **Combined yield** (isolated recrystallised material): **4 mg (2%)**.
^1H (acetone-d^6, 400 MHz): δ = 9.18 (br s, 1H, H6′), 8.29 (m, 1H, H3′), 8.22 (m, 1H, H4′), 8.11 (m, 1H, H4), 7.96 (m, 1H, H3/5), 7.84 (m, 1H, Ha/d), 7.78 (m, 1H, H3/5), 7.72 (m, 1H, H5′) 7.44 (m, 1H, Ha/d), 7.08-6.90 (m, 2H, Hb, Hc) ppm.

Cyclonickelation using $[Ni(HPhbpy)Br_2]_2$

Entry 7. This is the modified version of the reported procedure (NaO*t*Bu instead of KO*t*Bu).[10] $[Ni(HPhbpy)Br_2]_2$ (66 mg, 0.146 mmol) and NaO*t*Bu (28 mg, 0.292 mmol) were placed into 25-mL Schlenk flask. The flask was evacuated and back-filled with nitrogen for three times. Distilled Et_2O (15 mL) was added under stirring and nitrogen. The mixture was stirred for 24 h at ambient temperature. Evaporation left a beige material. No indication for the formation of the product was found from ^1H NMR, the starting material $[Ni(HPhbpy)Br_2]_2$ was recovered.

Entry 8. In the glovebox $[Ni(HPhbpy)Br_2]_2$ (104 mg, 0.23 mmol), KOAc (45 mg, 0.46 mmol), and K_2CO_3 (190 mg, 1.38 mmol) were placed in a 100 mL Schlenk flask. The flask was sealed and taken outside of the glovebox, distilled toluene (ca. 50 mL) was added under nitrogen. The mixture was refluxed using a Dean-Stark head under nitrogen for 15 h. Initially yellow suspension turned to intense red. Toluene (40 mL) was evaporated to Dean-Stark trap in the final hour of the reflux. The rest of the solvent was evaporated under vacuum. The yellow/brown solid was dissolved in CH_2Cl_2 (10 mL). The red solution was obtained after the filtration on Celite in air. The material was crystallised by the vapour diffusion of diethyl ether at room temperature. After 4 days, the red crystals with yellow, white, and green powders were obtained. The material was washed with diethyl ether (2 × 1 mL), methanol (quickly, 3 × 1 mL), and diethyl ether (2 × 1 mL). Red crystals were obtained. Yield: 6% (5 mg).

Entry 9. In the glovebox $[Ni(HPhbpy)Br_2]_2$ (65 mg, 0.14 mmol), KOAc (28 mg, 0.28 mmol), and K_2CO_3 (116 mg, 0.84 mmol) were placed into the 100 mL Schlenk flask. The flask was sealed and taken outside of the glovebox, distilled toluene (ca. 40 ml) was added under nitrogen. The mixture was refluxed with Dean-Stark head under nitrogen for 62 h. The initially yellow suspension turned to intense red. Toluene (15 mL) was evaporated to Dean-Stark trap in the final hour of the reflux. The rest of the solvent was evaporated under vacuum. The dark red solid was dissolved in CH_2Cl_2 (15 mL). The red solution was obtained after the filtration on Celite in air. The material was crystallised by the vapour diffusion of diethyl ether at room temperature. After 3 days, the red needle-like crystals were obtained. The material was washed with diethyl ether (2×1 mL), methanol (quickly, 1 × 1 mL), and diethyl ether (3 × 1 mL). Yield: 28% (15 mg).

Cyclonickelation using nickel(II) acetate

Entry 10. $Ni(OAc)_2 \cdot 4H_2O$ (129 mg, 0.520 mmol) was dissolved in ethanol (15 mL) and DME (20 mL) was added. The nitrogen-purged solution was added to the solution of HPhbpy (120 mg, 0.517 mmol) in distilled toluene (ca. 120 mL). The mixture was put into the oil bath (145°C), during 1 h low-boiling solvents (110 mL) were evaporated and eliminated using a Dean-Stark head. The mixture was stirred overnight for 15 h, the rest of the solvents evaporated during this time. There were red-black residues at the bottom and white solid on the walls of the flask. The crude material was washed by distilled toluene (1 × 20 mL). The toluene washings were concentrated giving white and red solid (89 mg) containing mainly the HPhbpy. The washed crude solid was suspended in distilled THF (ca. 50 mL), stirred for 30 min, filtered via cannula and concentrated. Crystallisation (THF/pentane, nitrogen, –20°C) gave red blocks of [Ni(Phbpy)(OAc)] suitable for XRD (see below) as well as side-off black and white material. The attempt to measure ^1H NMR spectrum of red crystals in either CD_2Cl_2 was unsuccessful. Red crystals degraded on air and in CH_2Cl_2 preventing further purification.

Entry 11. Anhydrous $Ni(OAc)_2$ (125 mg, 0.706 mmol) and HPhbpy (164 mg, 1 mmol) were heated using a heat gun under nitrogen (~250°C). After HPhbpy had melted, the colour started to change to red and turned to intense red in a few minutes. The addition of AcOH (10 mL) produced a green-yellow solution. The mixture in acetic acid was heated under reflux for 65 h then the solvent was evaporated leaving a green-grey product. The targeted complex [Ni(Phbpy)(OAc)] was not obtained.

Entry 12. $Ni(OAc)_2 \cdot 4H_2O$ (ca. 140 mg, 0.565 mmol) was dried at 90°C in vacuo for 15 h. HPhbpy (131 mg, 0.565 mmol, 1 eq) was added. The mixture was stirred at 180°C for 60 min under nitrogen: the colour changed from white-green to dark red and some black precipitate formed. The volatiles were evaporated *in vacuo* at 180°C. The solid was extracted using distilled THF (3 × 10 mL; and filtered (cannula, black precipitate separated) to the flask with NaCN (27 mg) in distilled THF (20 mL). The mixture was stirred for 2 h. NaCN was not dissolved by that time. Water (ca. 20 mL) was added to obtain a homogeneous solution. After 15 h agitation at ambient temperature the red colour of the solution was still present and the solvents were evaporated. The red crystals suitable for X-ray diffraction were obtained by vapour diffusion of diethyl ether to the CH_2Cl_2 solution. Yield: a few red crystals of [Ni(Phbpy)(CN)] (~4 mg).

Entry 13-17. In the glovebox $[Ni(HPhbpy)Br_2]_2$ (65 mg, 0.14 mmol), KOAc (28 mg, 0.28 mmol), and K_2CO_3 (39 mg, 0.54 mmol) were placed into a 100 mL Schlenk flask. The flask was sealed, taken outside of the glovebox and 40 mL distilled solvents were added under nitrogen. The mixture was heated under reflux using a Dean-Stark head under nitrogen for 64 h. The initially yellow suspension turned to intense red. From time to time samples were taken from the reaction vessel and UV-vis absorption spectra allowed to calculate the yield.

Entry 18. In the glovebox anhydrous $NiBr_2$ (30 mg, 0.14 mmol), HPhbpy, (33 mg, 0.14 mmol), KOAc (14 mg, 0.14 mmol), and K_2CO_3 (39 mg, 0.28 mmol) were placed into a 100 mL Schlenk flask. The flask was sealed and taken outside of the glovebox, 40 mL distilled *p*-xylene were added under nitrogen. The mixture was heated under reflux using a Dean-Stark head under nitrogen for 64 h. The initially yellow suspension turned to intense red. From time to time samples were taken from the reaction vessel and UV-vis absorption spectra allowed to calculate the yield.

Entry 19. In the glovebox $[Ni(HPhbpy)Br_2]_2$ (65 mg, 0.14 mmol) were placed into a 100 mL Schlenk flask. The flask was sealed and taken outside of the glovebox, 40 mL distilled 1,2-dichlorobenzene was added under nitrogen and the mixture was heated under reflux using a Dean-Stark head under nitrogen for 64 h. The initially yellow suspension turned slowly to red. From time to time samples were taken from the reaction vessel and UV-vis absorption spectra allowed to calculate the yield.

2. SUPPORTING FIGURES AND TABLES

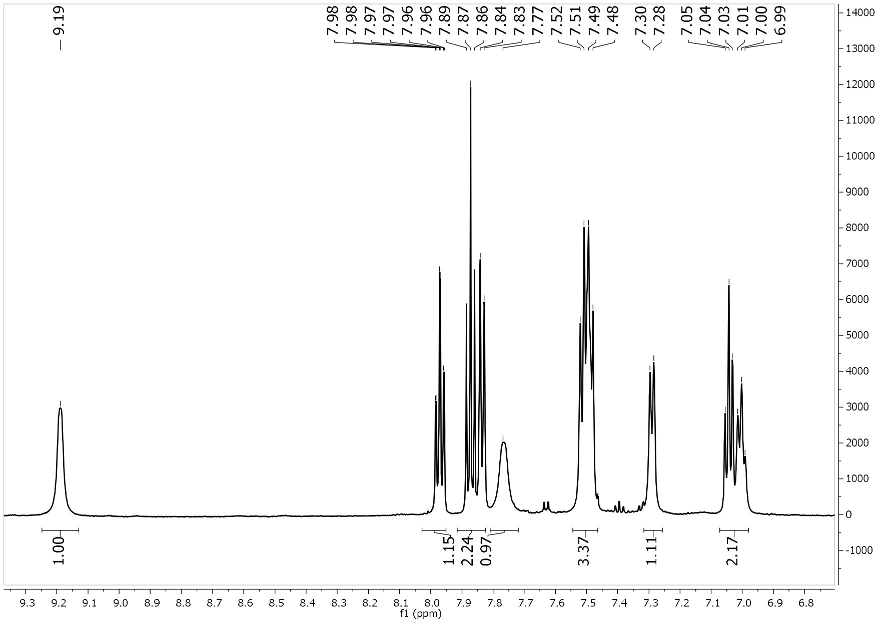

Figure S1. 600 MHz ^{1}H NMR spectrum of [Ni(Phbpy)Br] in CD_2Cl_2.

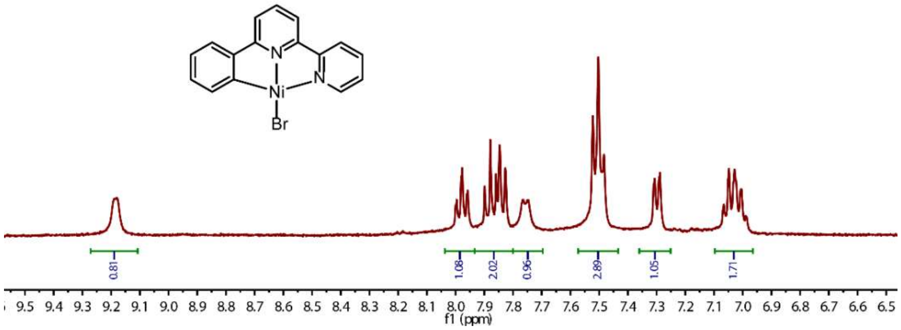

Figure S2. 400 MHz ^{1}H NMR spectrum of [Ni(Phbpy)Br] in CD_2Cl_2.

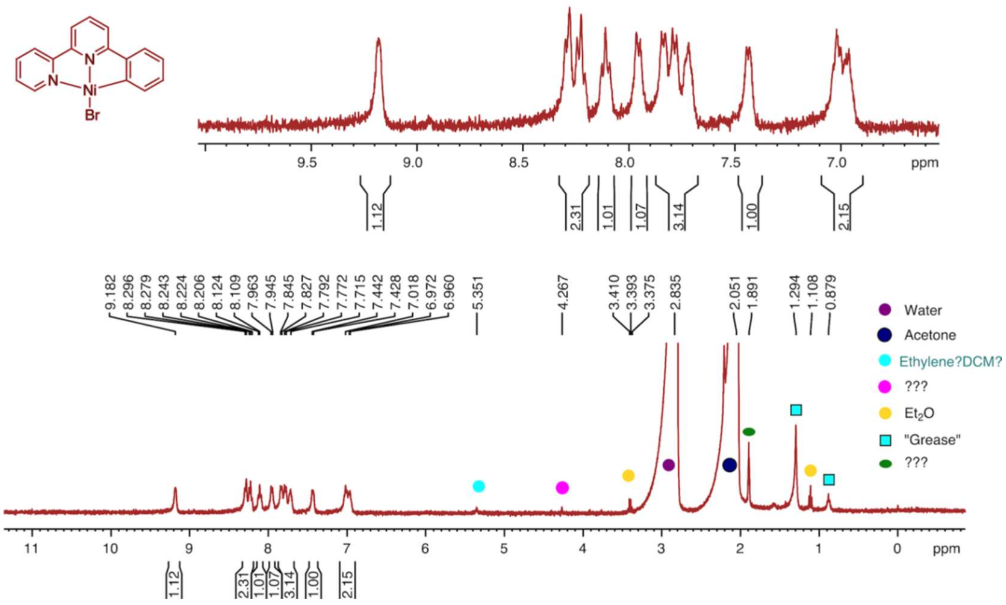

Figure S3. 400 MHz ^{1}H NMR spectrum of [Ni(Phbpy)Br] in acetone-d^6.

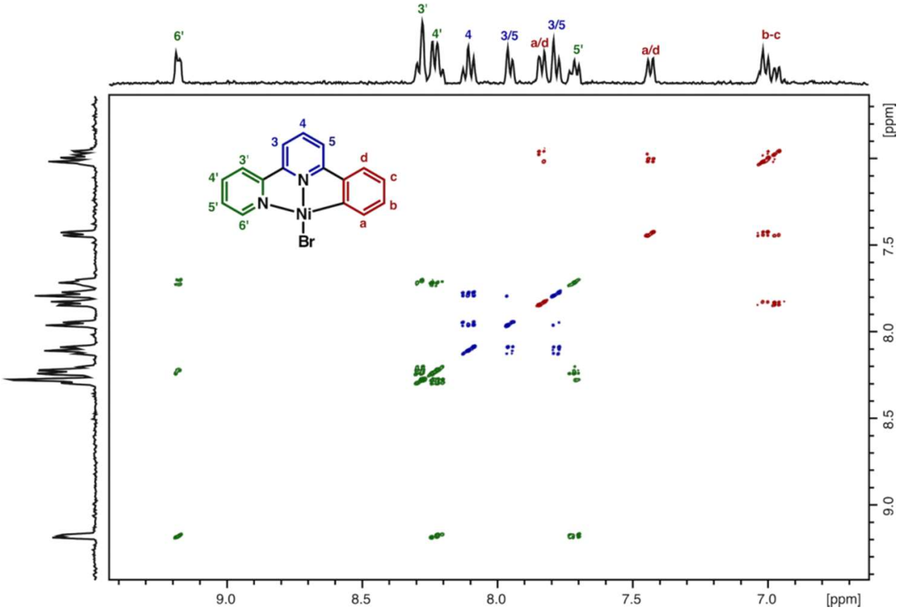

Figure S4. 400 MHz ^{1}H-^{1}H COSY spectrum of [Ni(Phbpy)Br] in acetone-d^6.

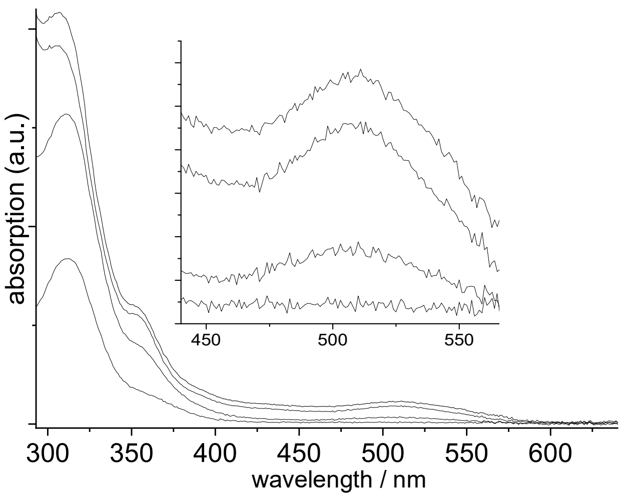

Figure S5. UV-vis absorption spectra recorded during the reaction of $[Ni(HPhbpy)Br_2]_2$ in *p*-xylene in the presence of $KOAc/K_2CO_3$, the spectra represent 0, 20, 80 and 100% yield.

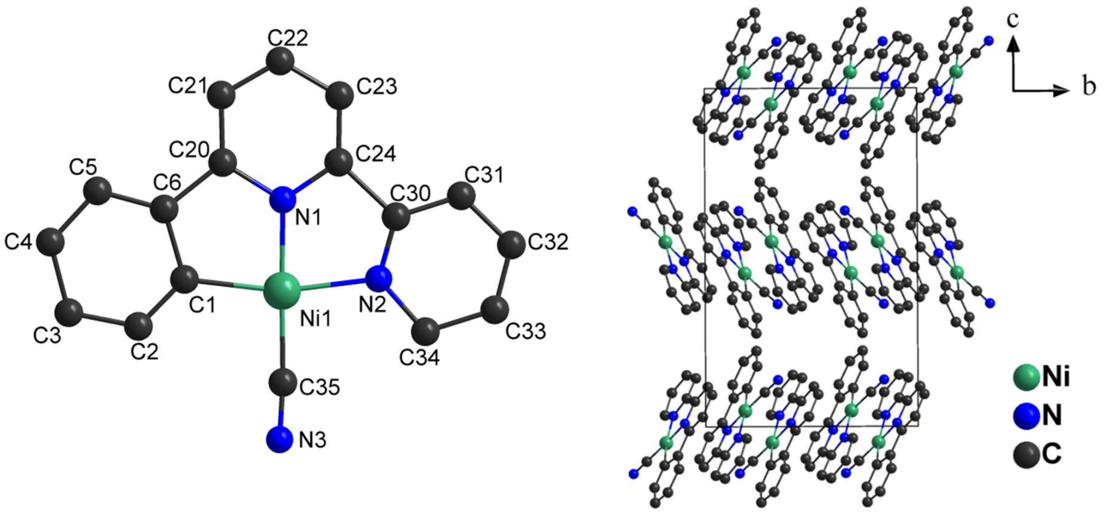

Figure S6. Molecular structure of [Ni(Phbpy)(CN)] (left) and crystal structure viewed along the *a* axis (right). Note that the structure could not be solved satisfactorily. These plots show only preliminary results.

Single crystal structure analysis (XRD) of $[Ni(HPhbpy)Br_2]_2$, [Ni(Phbpy)(OAc)], and [Ni(Phbpy)(CN)]

The red block crystals were obtained by diffusion of ether into a CH_2Cl_2 complex solution ($[Ni(HPhbpy)Br_2]_2$) or by gas phase diffusion of pentane to THF solution at −20 °C for [Ni(Phbpy)(OAc)] and [Ni(Phbpy)(CN)] and collected crystals were immersed in cryo-oil and mounted in a Nylon loop. Measurement of $[Ni(HPhbpy)Br_2]_2$ was performed at 170(2) K using an IPDS IIT (STOE and Cie.) diffractometer, with Mo-Kα radiation (λ = 0.71073 Å) employing ω-φ-2θ scan technique. The structure was solved by direct methods using SIR 2014[11] and refinement was carried out with SHELXL 2018 employing full-matrix least-squares methods on $F_0^2 \geq 2\sigma(F_0^2)$.[12] The numerical absorption corrections (X-RED V1.22; Stoe & Cie, 2001) were performed after optimising the crystal shapes using X-SHAPE V1.06 (Stoe & Cie, 1999). The non-hydrogen atoms were refined with anisotropic displacement parameters without any constraints. The hydrogen atoms were included by using appropriate riding models. Data of the structure solutions and refinements can be obtained free of charge at https://summary.ccdc.cam.ac.uk/structure-summary-form or from the Cambridge Crystallographic Data Centre, 12 Union Road, Cambridge, CB2 1EZ UK (fax: +44-1223 336033 or e-mail: deposit@ccdc.cam.ac.uk). For [Ni(Phbpy)(OAc)] and [Ni(Phbpy)(CN)] the X-ray diffraction data were

collected at 150(2) K with Bruker Kappa Apex-II diffractometer, using Mo-Kα radiation (λ = 0.71073 Å). The APEX2[13] program package was used for cell refinements and data reduction. Using Olex2,[14] the structures were solved with the SHELXS program using direct methods and refined with the ShelXL refinement package using least squares minimisation.[15] A numerical absorption correction (SADABS)[16] was applied to all data. For [Ni(Phbpy)(OAc)] the non-hydrogen atoms were refined with anisotropic displacement parameters without any constraints. All H atoms were positioned geometrically and constrained to ride on their parent atoms, with C−H = 0.95−0.99 Å and U_{iso} = 1.2−1.5U_{eq} (parent atom). The crystallographic details are summarised in Table S4. In contrast to this, the solution and refinement for [Ni(Phbpy)(CN)] remains preliminary, which is due to very low quality of the crystals (Table S5). At the same time, the crystalline material gave EI-MS showing the expected molecular footprint with an m/z of 316 corresponding to $[M+H]^+$.

Table S2. Crystal and refinement parameters of the complex $[Ni(HPhbpy)Br_2]_2$ and important structure parameters

Empirical formula	$C_{16}H_{12}Br_2N_2Ni_1$	bond lengths / Å	
Formula weight/g/mol	450.08	N1–Ni1	2.040(7)
Temperature/K	170(2)	N2–Ni1	2.040(7)
Crystal system	monoclinic	Ni1–Br1	2.604(1)
Space group	$P2_1/c$	Ni1–Br2	2.441(1)
a/Å	7.8572(5)	Ni1–(μ)Br1	2.483(1)
b/Å	19.136(2)	H1–Ni1	2.478(1)
c/Å	10.5205(7)	H1–Br1	2.886(1)
β/°	106.066(5)	C6–C20	1.47(1)
Volume/Å^3 / Z	1520.0(2) / 2	C24–C30	1.47(1)
ρ_{calc}g/cm^3	1.970	angles / °	
μ/mm^{-1}	6.526	N1–Ni1–N2	81.5(3)
F(000)	880	Br1–Ni1–Br2	167.16(5)
Crystal size/mm^3	0.1 x 0.1 x 0.1	N1–Ni1-Br1	87.8(2)
Radiation	MoKα (λ = 0.71073)	N2–Ni1–Br1	90.72
2Θ range for data collection/°	2.129 to 26.824	N1–Ni1-Br2	93.8(2)
Index ranges	$-9 \le h \le 9$, $-24 \le k \le 24$, $-13 \le l \le 12$	N2–Ni1-Br2	102.1(2)
Reflections collected	34159	N1–Ni1–(μ)Br1	170.0(2)
Independent reflections	3230 [R_{int} = 0.1991]	N2–Ni1–(μ)Br1	103.5(2)
Data/restraints/parameters	3230 / 0 / 191	N1–C30–C20–C1	149(1)
Goodness-of-fit on F^2	0.929	N1–C24–C30–N2	4(1)
Final R indexes [I>=2σ (I)]	R_1 = 0.0559, wR_2 = 0.1317		
Final R indexes [all data]	R_1 = 0.1025, wR_2 = 0.1538		
Largest diff. peak/hole / e Å^{-3}	0.996 / –1.065		
CCDC	1956740		

Table S3. Crystal data and structure refinement for [Ni(Phbpy)(OAc)].

Empirical formula	$C_{18}H_{14}N_2NiO_2$	Bond lengths / Å	
Formula weight/g/mol	349.02	C1–Ni1	1.90(1)
Temperature/K	150(2)	N1–Ni1	1.84(1)
Crystal system	monoclinic	N2–Ni1	1.97(1)
Space group	$P2_1/n$	O1–Ni1	1.87(1)
a/Å	12.9903(5)	C6–C7	1.461(2)
b/Å	7.8619(3)	C11–C12	1.469(2)

c/Å	14.7616(5)		
β/°	94.376(1)	angles / °	
Volume/Å^3 / Z	1503.2(1) / 4	C1–Ni1–N1	83.9(6)
ρ_{calc}g/cm^3	1.542	N1–Ni1–N2	82.21(5)
μ/mm^{-1}	1.301	N2–Ni1–O1	99.72(5)
F(000)	720.0	O1–Ni1–C1	94.18(6)
Crystal size/mm^3	0.537 × 0.339 × 0.264	C1–C6–C7–N1	0.4(2)
Radiation	MoKα (λ = 0.71073)	N1–C11–C12–N2	2.7(2)
2Θ range for data collection/°	5.536 to 64		
Index ranges	–18 ≤ h ≤ 19, –10 ≤ k ≤ 11, –21 ≤ l ≤ 22		
Reflections collected	34651		
Independent reflections	5199 [R_{int} = 0.0306, R_{sigma} = 0.0210]		
Data/restraints/parameters	5199/0/209		
Goodness-of-fit on F^2	1.013		
Final R indexes [I>=2σ (I)]	R_1 = 0.0313, wR_2 = 0.0807		
Final R indexes [all data]	R_1 = 0.0431, wR_2 = 0.0866		
Largest diff. peak/hole / e Å^{-3}	0.84 / –0.24		
CCDC	1956739		

Table S4. Crystal data and structure refinement for [Ni(Phbpy)(CN)].

Empirical formula	$C_{17}H_{11}N_3Ni$
Formula weight/g/mol	315.99
Temperature/K	150(2)
Crystal system	monoclinic
Space group	C2/c
a/Å	13.157(3)
b/Å	11.317(3)
c/Å	18.175(6)
β/°	97.582(6)
Volume/Å^3 / Z	2682(1) / 8
ρ_{calc}g/cm^3	1.496
μ/mm^{-1}	1.434
F(000)	1202
Crystal size/mm^3	0.1 x 0.1 x 0.1
Radiation	MoKα (λ = 0.71073)
2Θ range for data collection/°	2.261 to 28.135
Index ranges	–17 ≤ h ≤ 16, –14 ≤ k ≤ 14, –21 ≤ l ≤ 23
Reflections collected	14809
Independent reflections	3149 [R_{int} = 0.5471]
Data/restraints/parameters	3149 / 0 / 90
Goodness-of-fit on F^2	1.105
Final R indexes [I>=2σ (I)]	R_1 = 0.3643, wR_2 = 0.6713
Final R indexes [all data]	R_1 = 0.4414, wR_2 = 0.7011
Largest diff. peak/hole / e Å^{-3}	0.996 / –1.065
CCDC	Not submitted

3. REFERENCES

(1) P. Yang, Y. Yang, C. Zhang, X. J. Yang, H. M. Hu, Y. Gao, B Wu, Synthesis, Structure, and Catalytic Ethylene Oligomerization of nickel(II) and cobalt(II) Complexes with Symmetrical and Unsymmetrical 2,9-Diaryl-1,10-Phenanthroline Ligands, *Inorg. Chim. Acta* 2009, **362**, 89–96.

(2) D. L. Davies, S. A. Macgregor, C. L. McMullin, Computational Studies of Carboxylate-Assisted C−H Activation and Functionalization at Group 8−10 Transition Metal Centers, *Chem. Rev.* 2017, **117**, 8649−8709.

(3) J. F. Hartwig, *Organotransition Metal Chemistry: From Bonding to Catalysis*; University Science Books, 2010, p. 9.

(4) M. P. Feth, A. Klein, H. Bertagnolli, Investigation of the ligand exchange behaviour of square planar nickel(II) complexes by X-ray absorption spectroscopy and X-ray diffraction, *Eur. J. Inorg. Chem.* 2003, **2003**, 839–852.

(5) S. Sripothongnak, N. Barone, C. J. Ziegler, C–H bond activation and ring oxidation in nickel carbahemiporphyrazines, *Chem. Commun.* 2009, **30**, 4584–4586.

(6) A. Klein, B. Rausch, A. Kaiser, N. Vogt, A. Krest, The cyclometalated nickel complex [(Phbpy)NiBr] (Phbpy− = 2,2′-bipyridine-6-phen-2-yl) - synthesis, spectroscopic and electrochemical studies, *J. Organomet. Chem.* 2014, **774**, 86–93.

(7) A. Sandleben, N. Vogt, G. Hörner, A. Klein, On the Redox Series of Cyclometalated Nickel Complexes $[Ni((R)Ph(R')bpy)Br]^{+/0/-/2-}$ (H–(R)Ph(R′)bpy = substituted 6-phenyl-2,2′-bipyridine), *Organometallics* 2018, **37**, 3332–3341.

(8) L. S. Jongbloed, D. García-López, R. van Heck, M. A. Siegler, J. J. Carbó, J. I. van der Vlugt, Arene $C(sp^2)$-H Metalation at Ni^{II} Modeled with a Reactive PONCPh Ligand. *Inorg. Chem.* 2016, **55**, 8041–8047.

(9) R. A. Jagtap, V. Soni, B. Punji, Expeditious and Solvent-Free Nickel-Catalyzed C−H Arylation of Arenes and Indoles, *ChemSusChem* 2017, **10**, 2242–2248.

(10) E. C. Volpe, A. R. Chadeayne, P. T. Wolczanski, E. B. Lobkovsky, Heterolytic CH-bond activation in the synthesis of $Ni\{(2\text{-aryl-}\kappa C^2)\text{pyridine-}\kappa N\}_2$ and derivatives, *J. Organomet. Chem.* 2007, **692**, 4774–4783.

(11) M. C. Burla, R. Caliandro, B. Carrozzini, G. L. Cascarano, C. Cuocci, C. Giacovazzo, M. Mallamo, A. Mazzone, G. Polidori, Crystal structure determination and refinement via SIR2014. *J. Appl. Crystallogr.* 2015, **48**, 306–309.

(12) G. M. Sheldrick. SHELXL 2016. *Acta Crystallogr., Sect. A: Found. Crystallogr.* 2008, **64**, 112–122.

(13) APEX2 − Software Suite for Crystallographic Programs. *Bruker AXS, Inc.*, Madison, WI, USA, 2010.

(14) O V. Dolomanov, L. J. Bourhis, R. J. Gildea, J. A. K. Howard, H. Puschmann, OLEX2: A Complete Structure Solution, Refinement and Analysis Program. *J. Appl. Crystallogr.* 2009, **42**(2), 339–341.

(15) G. M. Sheldrick, Crystal Structure Refinement with SHELXL. *Acta Crystallogr. Sect. C: Struct. Chem.* 2015, **71**(1), 3–8.

(16) G. M. Sheldrick, SADABS-2008/1 − Bruker AXS Area Detector Scaling and Absorption Correction. *Bruker AXS, Inc.*, Madison, WI, USA, 2008.
